# What do school personnel know, think and feel about food allergies?

**DOI:** 10.1186/2045-7022-3-39

**Published:** 2013-11-25

**Authors:** Laura Polloni, Francesca Lazzarotto, Alice Toniolo, Giorgia Ducolin, Antonella Muraro

**Affiliations:** 1 Food Allergy Referral Centre Veneto Region, Department of Women and Child Health, Padua General University Hospital, Via Giustiniani, 3 - 35128 Padua, Italy

**Keywords:** School, Food allergy, Anaphylaxis

## Abstract

**Background:**

The incidence of food allergy is such that most schools will be attended by at least one food allergic child, obliging school personnel to cope with cases at risk of severe allergic reactions. Schools need to know about food allergy and anaphylaxis management to ensure the personal safety of an increasing number of students. The aim of this study was to investigate Italian school teachers and principals’ knowledge, perceptions and feelings concerning food allergy and anaphylaxis, to deeply understand how to effectively support schools to manage a severely allergic child. In addition a further assessment of the impact of multidisciplinary courses on participants was undertaken.

**Methods:**

1184 school teachers and principals attended courses on food allergy and anaphylaxis management at school were questioned before and after their course. Descriptive and inferential statistics were used to analyze the resulting data.

**Results:**

Participants tended to overestimate the prevalence of food allergy; 79.3% were able to identify the foods most likely involved and 90.8% knew the most frequent symptoms. 81.9% were familiar with the typical symptoms of anaphylaxis but, while the majority (65.4%) knew that “adrenaline” is the best medication for anaphylaxis, only 34.5% knew indications of using adrenaline in children. 48.5% thoroughly understood dietary exclusion. School personnel considered that food allergic students could have social difficulties (10.2%) and/or emotional consequences (37.2%) because of their condition. “Concern” was the emotion that most respondents (66.9%) associated with food allergy. At the end of the course, the number of correct answers to the test increased significantly.

**Conclusions:**

Having adequately trained and cooperative school personnel is crucial to significantly reduce emergencies and fatal reactions. The results emphasize the need for specific educational interventions and improvements in school health policies to support schools to deal with allergic students ensuring their safety and psychological well-being.

## Background

The incidence of food allergies (FA) in children has increased in recent years [1]. It has been estimated that FA affects up to 4-7% primary school children in Europe [2]. The lifetime prevalence of parental perceived allergic reactions to food was 10.5% in Italian school-aged children [3]. The prevalence of FA is such that most schools will be attended by at least one child with FA, obliging school personnel to cope with children at risk of severe allergic reactions. It has been estimated, moreover, that between 10% and 18% of food allergy or anaphylaxis reactions occur at school [4]. In food allergic people aged 0–19 fatal food anaphylaxis was found to have an incidence rate of 3.25 per million person years [5]. Overall, anaphylaxis is a rare event in school-age children, but anaphylaxis and FA deaths have been reported at school [6]. School principals and teachers increasingly need to mediate the parents’ concern about their children having an allergic reaction with the everyday running of the school [7]. Numerous studies on FA and anaphylaxis treatment in school have identified some major shortcomings, such as the lack of proper FA management plans as well as inadequacies in recognizing and treating reactions with epinephrine [8,9]. It has been highlighted that schools need to formally educate staff on FA, providing information on prevention measures, establishing treatment plans and training staff to administer epinephrine, where appropriate [2,10-13]. Clearly, school teachers need to know about FA and anaphylaxis management to ensure the personal safety of an increasing number of school students.

It is worth mentioning also that there are no nurses on the school staff in Italy, so the management of food allergic students weighs on school personnel. The aim of this study was to investigate what school teachers and principals know, think and feel about FA and anaphylaxis in order to deeply understand how to help schools to effectively manage a severely allergic child. In addition the impact of the courses on participants was assessed comparing answers before and after the session.

## Methods

A total of 1184 Italian school teachers and principals, who voluntarily attended free courses on FA and anaphylaxis management at school took part in the study. Participants came from schools in the Veneto Region, from nursery to high school level, the majority coming from nursery and primary schools as shown in Table 1.

**Table 1:** Scores for correct answers before the course for different types of school

**Type of school**	**N respondents**	**Mean**	**Standard deviation**	**N**	**F**	**Df**	**P**
Nursery school (children aged 0–5)	295	6.83	1.727	1184	13.450	2	.001
Primary school (6–11)	598	7.07	1.646				
Middle and High school (12–19)	291	6.21	1.722				

A questionnaire was developed by a multidisciplinary team of experts in the field basing on clinical expertise and current literature data. The questionnaire was firstly given to some representatives of the target group in a pilot project, to test its clearness. It contained multiple-choice and open questions assessing knowledge, thoughts and feelings about FA and anaphylaxis. Questions are displayed in Table 2.

**Table 2:** Questions proposed to participants

**Questions assessing knowledge, thoughts and feelings about food allergy and anaphylaxis**	
Did you ever receive information about food allergy and anaphylaxis? □ yes □ no	
If so from which source? *	
□ First aid courses □ Health related training □ Mass media □ Web □ Other	
*Knowledge*	
1	How many patients suffer from food allergies in childhood?
	□ 1%
	□ 20-50%
	□ 4-7%
	□ 15%
2	What foods most likely cause food allergy?
	□ Tomato, strawberries, chocolate
	□ Milk, egg, fish, nuts, wheat
	□ Legumes, rice, potatoes
	□ Apple, spinach, carrot
3	What are the most frequent symptoms of food allergy?
	□ Urticaria, stomachache, wheezing
	□ Headache, fever, tremors
	□ Constipation, headache, nausea
	□ Conjunctivitis, tonsillitis
4	What are the most frequent symptoms of anaphylaxis?
	□ Asthma, dermatitis
	□ Conjunctivitis, rhinitis, headache
	□ Urticaria, itch, stomachache, wheezing, throat tightness, collapse
	□ Tonsillitis, cough, temperature
5	Which is the best medication for anaphylaxis?
	□ Orally antihistamine
	□ Cortisone
	□ Intramuscular adrenaline
	□ Intramuscular antihistamine
6	Are there any contraindications for using adrenaline in children in case of anaphylaxis?
	□ Diabetes
	□ Children under 3 years of age
	□ Drug hypersensitivity
	□ No contraindications
7	How can you assure food preparation is “safe” from allergens?
	□ Washing hands before cooking
	□ It is not possible
	□ Avoiding all possible cross-contaminations in the whole meal production
	□ Washing kitchen stuff before cooking
8	How can you assure if packaged food is “safe” from a specified allergen?
	□ Reading labels
	□ Tasting the food
	□ It is not possible
	□ Asking to someone
9	What does dietary exclusion consist of?
	□ Consuming only foods free from allergens
	□ Consuming only foods free from additives or preservatives
	□ Consuming only fresh food
	□ Consuming only home-made food
10	What are the possible risks of dietary exclusion?
	□ Stomachache
	□ No risks
	□ Undernourishment, social limitations
	□ Skin diseases
*Thoughts*	
In your opinion, food allergic students*	
□ Could have relationship difficulties because of their health conditions	
□ Could have academic difficulties because of their health conditions	
□ Could have emotional difficulties because of their health conditions	
□ Don’t have any social, academic or emotional difficulties because of their health conditions	
Do you think that food allergy and anaphylaxis could be managed at school by school personnel?	
□ Yes □ No	
Do you think that the management of food allergy and anaphylaxis at school is a task of the school personnel?	
□ Yes □ No	
In your opinion, what could be useful to manage food allergic students?**	
What do you think could be useful to support food allergic students?**	
In your opinion, what are the main difficulties in managing food allergy and anaphylaxis at school?**	
*Feelings*	
What emotions do you mainly feel about managing food allergy at school?*	
□ Anxiety	
□ Concern	
□ Fear	
□ Helplessness	
□ Other	

*more than one choice is possible.

**open question.

School workers were questioned anonymously in written form before and after their course. They were advised that their data would be used for the research aim of better understanding school needs in managing students at risk of anaphylaxis.

The courses were free of charge organized by the Veneto Food Allergy Centre in Padua over a 12 months period and consisted of one 2-hour intensive session conducted by a pediatric allergist, a dietician, a psychologist, and a lawyer. Courses assessed were 10 in total.

Descriptive statistics were used on pre-course data to explore the school teachers’ baseline knowledge, attitudes and feelings on the topic. For each item, the frequency and percentage of answers was calculated. For each participant, the frequency of correct answers to questions 1 to 10 was calculated, concerning what they knew about FA and anaphylaxis. The questions on their thoughts and feelings had no right or wrong answers, so it was only computed the frequency of the answers.

Inferential statistics were used to investigate the data collected. One-way analysis of variance and the Bonferroni post-hoc test were used to identify differences in the scores between the different types of schools, and the chi-square test was used to investigate differences in teachers’ opinions and feelings about FA, again by type of school. Data obtained before and after the course were then compared using the t-test and chi-square test to identify changes in participants’ answers. Findings were analyzed using the SPSS 17 statistical software package. The level of significance was set at .05.

## Results

The results showed that, among the study sample as a whole, 753 (63.6%) had already received information on FA: 71.7% attended first aid courses; 11.1% attended health related training; 64.5% had information from mass media, 23% from the web and 1.4% from other sources (e.g. acquaintances or relatives). However, this did not influence the number of correct answers in the baseline questionnaire (p < .05).

### Knowledge

At baseline, the following picture emerged regarding knowledge about FA: interestingly, 60.2% of participants overestimated food allergy prevalence in children. On a positive note respondents seem to know about food allergy: 79.3% were able to identify foods most likely to cause FA; 90.8% knew most of the frequent symptoms; and 81.9% were familiar with typical symptoms of anaphylaxis. However, while 65.4% knew that adrenaline is the best medication for anaphylaxis, only 34.5% knew that self-injectable adrenaline can be used in children without any risks of severe side effects. When it came to food preparation, 84.5% of teachers were aware that it was essential to prevent cross-contamination of food and 81.8% recognized the need to read food labels. However, only 48.5% knew what “dietary exclusion” means, and 60.4% could correctly identified the risks relating to dietary exclusion, e.g. undernourishment or social problems.

Total scores for primary schools were higher than nursery or secondary schools (F value (F) = 13.450, degree of freedom (df) = 2, p < .001), as assessed by one-way analysis of variance (Table 1) and confirmed by Bonferroni’s post-hoc test.

**Figure 1:**
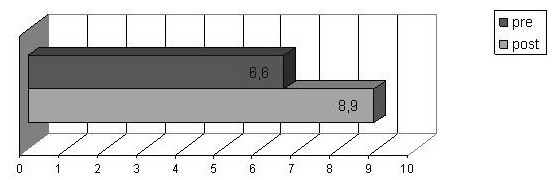
Knowledge on food and anaphylaxis: before and after course mean score.

The total scores obtained after the course were significantly better (t value (t) = −34.191, df = 2366, p < .001) and show an increase from a mean score of 6.6 (±1.755) to 8.9 (±1.340) as shown in Figure 1.

### Thoughts

**Figure 2:**
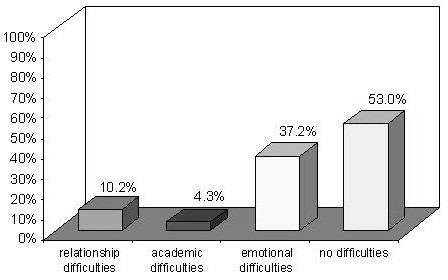
Difficulties expected to be caused by FA.

As for school teachers’ and principals’ thoughts about FA (Figure 2), the following figures emerged at baseline for the positive answers when they were asked if allergic students could have learning difficulties (4.3%), social difficulties (10.2%), or emotional consequences (37.2%) as a result of their allergy, while 53% thought that allergic students suffered no consequences of their FA at school.

**Figure 3:**
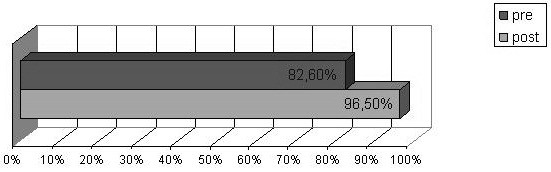
**Thoughts: could food allergy and anaphylaxis be managed at school by school personnel?** Yes answers percentage before and after the course.

**Figure 4:**
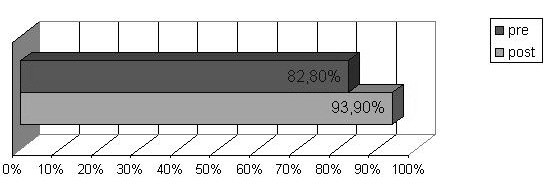
**Thoughts: is the management of FA and anaphylaxis at school a task of the school personnel?** Yes answers percentage before and after the course.

In the questionnaires completed before the course, 82.6% of participants considered that FA and anaphylaxis could be managed at school and 82.8% thought that this was the responsibility of the school personnel. Most participants (89.6%) reported that specific multidisciplinary courses were helpful to manage FA and anaphylaxis adequately at school. School personnel felt that the main difficulty in managing FA and anaphylaxis at school was: the lack of specific training (78.2%); the need to ensure that children with FA avoid allergens (14.7%); and the worry, anxiety and fear in the event of a FA-related emergency (7.1%). It was also considered crucially important to spend some time in class listening to allergic students’ issues, discussing them (49.3%), and developing the students’ abilities and potential (29.8%). There were no significant differences in the thoughts reported at the different types of school. The frequency of participants thinking that anaphylaxis could be managed at school (χ^2^ = 108.757, df = 1, p < .001) and that it is the responsibility of the school personnel (χ^2^ = 64.232, df = 1, p < .001) significantly increased after the course as shown in Figures 3 and 4.

### Feelings

**Figure 5:**
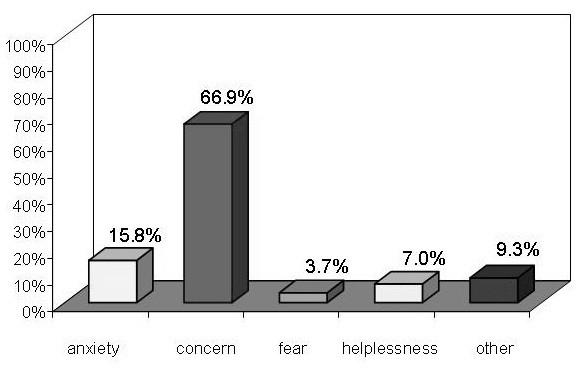
Feelings of school workers about FA.

When asked in the questionnaire about their feelings concerning FA at school (Figure 5), most participants (66.9%) said, at the baseline, the main emotion was “concern”; 15.8% of school workers reported “anxiety”; 3.7% mentioned “fear”; and 7% felt “helpless”. The answer to this question was “other” for 9.3% of teachers, which indicated a positive attitude, such as the hope that they would be able to deal with the allergy and the wish to do something useful. There were no significant differences in the feelings reported at the different types of school and after the course, even if a decreasing in the frequency of “concern” answers was registered.

## Discussion

It is worth noting that, although many of our respondents had already received some form of information on FA, it had no influence on the outcome of the questionnaire. Their knowledge came not only from mass media, but mostly from first aid courses and/or other health-related training, raising concern that some participants may have attended first-aid courses that failed to provide training on how to manage FA and anaphylaxis. Other studies have shown that, although the school system does its best to try and manage emergencies, when it comes to FA they are often very badly prepared [9,14]. This points out the need for both, the school and the health system, to focus on preparedness of school personnel to manage food allergic students.

The aim of this study was to investigate what school teachers and principals knew about FA and anaphylaxis, and their related thoughts and feelings. Regarding their *knowledge*, it is interesting to note that they overestimated the prevalence of FA in children: this might reflect increasing concern in schools about FA and their lack of preparation to deal with what is seen as a growing problem for schools [2]. It might also relate to the fact that “food allergy” is often misused as a generic label for food-related problems; for instance, as demonstrated, many people do not know the difference between “food allergy” and “intolerance” [15].

The present questionnaire identified an encouragingly high percentage of participants who were able to identify the commonly-involved foods and most frequent symptoms of FA and anaphylaxis correctly. On the other hand, a much lower proportion of them knew that adrenaline is the best medication for anaphylactic shock. The most worrying finding, however, was that only 34.5% of respondents knew there are no absolute contraindications to administer self-injectable adrenaline in children, which stands for many school teachers were reluctant to use it because of supposed side effects in childhood. It is a common myth that a life-saving drug can be harmful, and there is fear and mistrust surrounding the use of adrenaline, even though it has become well established as the best treatment for anaphylaxis, and endorsed by medical experts. A delay in administering epinephrine is a common factor associated with fatal outcomes of FA in children and adolescents [6]. The usage of self-injectable adrenaline is still quite low; it was highlighted the paucity of knowledge concerning when and how to use the device, and reported that adequate first-aid measures were not in place for the majority of school-going children [16,17]. Care-givers need to be trained continually and given support on first-aid anaphylaxis management [17,18]. Previous studies reported also that teachers have very limited knowledge about anaphylaxis. This observation prompts the need to inquire into the allergy management plans and policies in schools [17,18]. A comprehensive educational program for teachers is considered imperative when no school nurse is available [19].

Food preparation represents an essential issue when dealing with FA. The study questioned participants about their understanding of dietary requirements. The majority recognized the need to prevent cross-contaminations of food and the importance of reading food labels. Nonetheless, only 48.5% really knew what an exclusion diet is, and the questionnaire startlingly revealed that many respondents thought that an exclusion diet meant eating fresh or home-made food, with no additives or preservatives. Just over half of the teachers rightly acknowledged that children on exclusion diets might be at risk of nutritional deficiencies and/or social limitations. The latter issue relates to the fact that food has a social value because it is often associated with relationships. Going out with friends, eating in the canteen, even going to parties may pose problems for allergic children and adolescents, and/or their families, with fallout on their quality of life [7].

Primary schools had higher overall scores than nursery or secondary schools when it came to the teachers’ knowledge of FA: this may reflect the composition of our sample (the primary school teachers were numerically better represented). On the other hand, it may relate to the epidemiology of FA. It has been estimated that FA affects up to 4-7% of primary school children [2], so teachers of this age group are likely to be more aware of the problem and more motivated to obtain information on how to deal with the related problems.

A purpose of the questionnaire was to investigate what participants *thought* about FA and anaphylaxis. This was important with a view to establishing whether school teachers had any prejudiced or misconceived ideas about FA that might influence how the condition is managed at school. Only a very small percentage acknowledged that a student with FA could have academic difficulties. FA is often accompanied by respiratory allergies [20] and it has been demonstrated that respiratory diseases can affect a student’s performance [21]. Another significant issue is that students with allergies have to see doctors more often, meaning they are often absent from class and this influences their school results [22,23].

Only 10.2% of our respondents recognized that allergic students may suffer from relational difficulties, and 37.2% felt that they might have emotional problems. Although it has been demonstrated in the literature that allergic patients can have various severe relational and emotional difficulties [23], more than half of the participants believed that students with FA suffered no such consequences of this condition. Less than half of the teachers acknowledged the importance of creating opportunities in class for listening and sharing the students’ challenges. These findings give cause for concern, since they could mean that school teachers tend to underestimate some students’ important issues, and they are ill-prepared to manage the psychological issues associated with FA. Many of the respondents said that the main difficulty of managing FA at school was the lack of specific training, confirming previous research and experts’ reports [2,17,18]. Most of the teachers recognized that multidisciplinary courses are needed to manage all aspects of FA adequately. On the other hand, an encouraging finding was that most of the participants felt that FA and anaphylaxis *can* be managed at school, and that it is up to the teachers to do so, showing a proactive approach and willingness to do better. This is likely linked also to the fact that Italian school health policy does not employ school nurses. In the case of managing FA at school Veneto Region had a law in place that recommends the collaboration among schools, patients’ families, health professionals and local health services. Specific training for school personnel is required, but not mandatory. The findings of the study highlighted the need for policy changes and reform to support and empower the school system in adequately managing food allergic students.

The final part of the questionnaire focused on how the teachers *felt* about managing FA and anaphylaxis at school. It is important to understand their feelings to ensure their full cooperation in managing FA. It is common knowledge that anxiety and fear can make people freeze in an emergency situation; these feelings can also lead to unnecessarily restrictive school environments as well as affect health care planning, giving rise to conflicts within families and with physicians and the school community [24]. The main feeling reported by our participants was “concern”; only 15.8% mentioned “anxiety” and 7% felt “helpless”. Encouragingly, only 3.7% said they were fearful about FA. Even more positive is the fact that 9.3% mentioned “other” feelings, which they later described as the hope the child would recover from the allergy and their wish to do something useful to help allergy sufferers. The results did not differ for respondents working at different types of school (nursery, primary or secondary school teachers). Teachers’ attitudes have proved to be an important factor in ensuring appropriate treatment for allergic children [8,17]. In general, the findings showed an apprehension felt by school teachers relating to children with FA. These feelings were not seen as an obstacle and they could be managed and turned to positive account. It can be supposed they reflect the need of school to be better supported in managing food allergic students, for example considering the availability of school nurses or mandatory training programs. The management of FA in the school setting should also include providing resources for school officials to help them develop FA management protocols [24].

In addition, an assessment of the answers before and after the course was performed. When the questionnaire was completed again, there was a significant increase in the overall scores about knowledge and in the frequency of participants thinking that anaphylaxis could be managed at school and that it is the responsibility of the school personnel. A modest lessening in the frequency of “concern” answers was registered. Results confirmed a general positive effect of the course on the participants’ understanding of FA and anaphylaxis. As expected, changing personal opinions and feelings seems to need more time and efforts than modifying knowledge. Further and long-term studies are needed to know the effectiveness of multidisciplinary courses in terms of reducing the number of reactions and increasing food allergic students’ quality of life at school.

The study is descriptive in its nature and it depends mostly upon impressions from the questionnaire; however, this limitation could be balanced by the big large of the sample (1184 school workers), so results seem to be in any case informative. Another limit could be the fact that national differences in school policy do not allow for generalization of findings, however it can be useful to learn about and compare different practices for managing allergy and anaphylaxis with a view to improving regulations and guidelines for schools.

Results from Veneto Region could represent a starting point toward validation of multidisciplinary educational trainings that could be used on a larger scale with a benefit for all Italian schools. This is indeed the first study, as far as we know, assessing Italian school personnel attitudes toward the management of FA and anaphylaxis in the school setting. The gaps identified could form the basis for improvements of local and national legislation in order to ensure implementation of specific educational interventions for an adequate management of FA and anaphylaxis at school. Physician and Referral Health Centre can play an important role in educating school personnel about the treatment of food allergies [24]. As recommended by the European Academy of Allergology and Clinical Immunology [2], an education network involving health care and education providers is crucial in ensuring that the school staff is alerted and trained, and specific allergy management plans initiated. This should be achieved through the empowerment of key stakeholders and supported by continuing education of all school staff.

## Conclusions

Managing FA and anaphylaxis demands major efforts and changes in the school system: it is crucial to have adequately informed, trained and cooperative school staff to significantly reduce the incidence of emergencies and fatal allergic reactions. Understanding the schools’ needs and attitudes is the first essential step to the success of any training scheme. The results highlight areas where there is a lack of not only knowledge, but also understanding of the students with food allergies in terms of their well-being as well as their risk of having a reaction. A positive effect on school personnel’s knowledge and thoughts after the course was registered. This contributes to underline the role of specific educational programs to train school teachers to deal with allergic children but also the necessity of implementing an adequate and comprehensive school health policy to ensure the safety and well-being of an increasing number of students.

## Abbreviations

Df: Degree of freedom; FA: Food allergy; F: F value; t: t value.

## Competing interests

No financial support was awarded relating to this paper. Conflict of interest: nothing to declare.

## Authors’ contributions

LP designed the study, collected and analyzed the data and prepared the manuscript. FL, AT and GD collected the data, provided intellectual input and assisted in manuscript preparation. AM coordinated and supervised the study, provided intellectual input and assisted in manuscript preparation. All authors read and approved the final manuscript.
